# Cell size matters: CDKG2 regulates endoreduplication in Arabidopsis

**DOI:** 10.1093/plcell/koac029

**Published:** 2022-01-31

**Authors:** Sophie Hendrix

**Affiliations:** Assistant Features Editor, The Plant Cell, American Society of Plant Biologists, USA; Institute of Crop Science and Resource Conservation, University of Bonn, Bonn, Germany; Centre for Environmental Sciences, Hasselt University, Diepenbeek, Belgium

To visualize individual plant cells, researchers typically rely on microscopes. However, some cell types grow so large that they can be distinguished with the naked eye. An example is Arabidopsis trichomes, which are single cells with a typical branched structure, found on the leaf surface. These specialized cells depend on multiple rounds of endoreduplication to reach their impressive size. Endoreduplication (also known as endoreplication) is an alternative version of the classical cell cycle during which cells replicate their nuclear DNA without subsequently dividing, thereby increasing their ploidy level. This process occurs in a variety of cell types and contributes to the normal growth and development of most herbaceous angiosperms. Furthermore, it serves a role in plant responses to biotic and abiotic stresses (Lang and Schnittger, 2020). However, our understanding of the molecular mechanisms underlying this cell cycle variant is far from complete. Previously, UBIQUITIN-SPECIFIC PROTEASE14 (UBP14), encoded by the *DA3* gene, was identified as a negative regulator of endoreduplication in Arabidopsis. Plants with a partial loss-of-function mutation in *DA3* are characterized by enlarged cotyledons with an increased cell size and higher ploidy levels. Furthermore, their leaves harbor trichomes with a higher number of branches in comparison to wild-type plants (Xu et al., 2016).

In this issue, **Shan Jiang and colleagues (****Jiang et al., 2022****)** show that the cyclin-dependent kinase CDKG2 regulates endoreduplication downstream of UBP14 in Arabidopsis (see Figure). In a genetic screen, they discovered that the *suppressor of da3 6* (*sud6*) mutation partially suppressed the *da3* phenotype. Using a mapping approach, they pinpointed this mutation to CDKG2, which was previously shown to play a role in thermo-sensitive splicing of a gene modulating flowering time (Nibau et al., 2020). Microscopic and flow cytometric investigation of *sud6* single mutants revealed that their cotyledon cells were smaller and had lower ploidy levels when compared with those of wild-type plants, whereas the opposite was observed for *SUD6*-overexpressing plants. Together, these data suggested that CDKG2 positively regulates endoreduplication, which was further supported by the nuclear localization of the CDKG2-GFP fusion protein.

**Figure:**
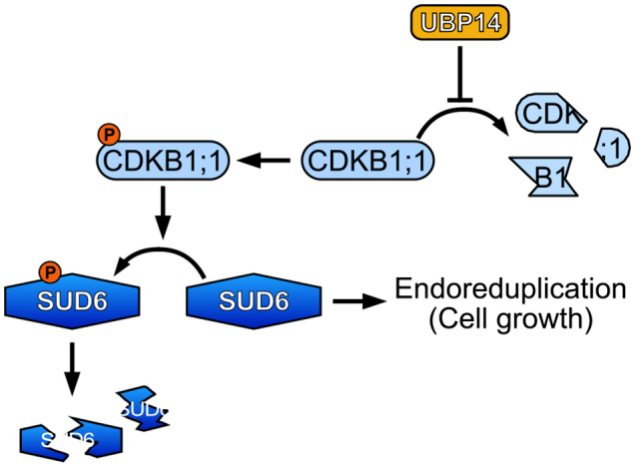
Schematic overview of UBP14-CDKB1;1-SUD6-mediated regulation of endoreduplication in Arabidopsis. DA3/UBP14 influences the abundance of CDKB1;1, which phosphorylates SUD6/CDKG2 and reduces its stability. SUD6/CDKG2 positively regulates endoreduplication through an unknown mechanism. Reprinted from Jiang et al. (2022), Figure 7.

Next, the interaction of CDKG2 with CDKB1;1 was investigated, as previous work by the authors showed that UBP14 modulates the stability of CDKB1;1 and its associated cyclin CYCA2;3 (Xu et al., 2016). The *sud6* mutation partially rescued the cotyledon area, cell size, and ploidy levels of a dominant negative *CDKB1;1* mutant, suggesting that CDKG2 operates downstream of CDKB1;1. Using a combination of in vitro and in vivo approaches, Jiang et al. (2022) showed that CDKG2 physically interacts with CDKB1;1 but not CYCA2;3. As CDKs can phosphorylate their substrates even in the absence of their cyclin partners, the authors next looked into the phosphorylation state of CDKG2. Phosphorylation assays using the recombinant proteins revealed that CDKB1;1 phosphorylates CDKG2 and these results were confirmed in vivo. CDKG2 phosphorylation likely reduces its stability, as CDKG2 protein levels were significantly increased in the dominant negative *CDKB1;1* mutant.

In conclusion, this work by Jiang et al. (2022) clearly demonstrates the involvement of CDKG2 in endoreduplication in Arabidopsis. However, the molecular mechanism underlying CDKG2-mediated increases in cellular ploidy levels remains elusive. In this context, the previously demonstrated role of CDKGs in alternative splicing should be considered, as many cell cycle genes were differentially expressed in the *sud6* background. While the splicing of these cell cycle genes was unaffected, it is tempting to speculate that CDKG2 induces alternative splicing of transcription factors controlling their expression. Another challenge that remains is the identification of cyclins associated with CDKG2 in endoreduplication regulation. Undoubtedly, this work forms an excellent starting point to further explore the role of CDKG2 in this fascinating process.
